# Plant interaction modifies effects of soil heterogeneity on seed germination, plant growth, and biomass of plant communities

**DOI:** 10.1093/aobpla/plaf013

**Published:** 2025-03-08

**Authors:** Hui Li, Yumao Ning, Mingrui Liu, Shiting Liu, Yongjie Liu

**Affiliations:** State Key Laboratory of Herbage Improvement and Grassland Agro-ecosystems; Key Laboratory of Grassland Livestock Industry Innovation, Ministry of Agriculture and Rural Affairs; College of Pastoral Agriculture Science and Technology, Lanzhou University, Lanzhou, China; State Key Laboratory of Herbage Improvement and Grassland Agro-ecosystems; Key Laboratory of Grassland Livestock Industry Innovation, Ministry of Agriculture and Rural Affairs; College of Pastoral Agriculture Science and Technology, Lanzhou University, Lanzhou, China; State Key Laboratory of Herbage Improvement and Grassland Agro-ecosystems; Key Laboratory of Grassland Livestock Industry Innovation, Ministry of Agriculture and Rural Affairs; College of Pastoral Agriculture Science and Technology, Lanzhou University, Lanzhou, China; State Key Laboratory of Herbage Improvement and Grassland Agro-ecosystems; Key Laboratory of Grassland Livestock Industry Innovation, Ministry of Agriculture and Rural Affairs; College of Pastoral Agriculture Science and Technology, Lanzhou University, Lanzhou, China; State Key Laboratory of Herbage Improvement and Grassland Agro-ecosystems; Key Laboratory of Grassland Livestock Industry Innovation, Ministry of Agriculture and Rural Affairs; College of Pastoral Agriculture Science and Technology, Lanzhou University, Lanzhou, China

**Keywords:** neighbour effect, patch size, plant height, plant biomass, soil heterogeneity

## Abstract

Soil heterogeneity significantly impacts the structure and function of plant communities. However, most of the previous studies only focussed on the effects of soil heterogeneity on plant populations, while the joint effects of plant interaction and soil heterogeneity on plant communities remain unclear. Thus, a manipulation experiment was done to explore the effects of soil heterogeneity and species combination on the seed germination, plant height and plant biomass, where three soil heterogeneity levels were created by varying patch sizes (small, medium, and large), and 10 species combinations were generated by growing four typical forages on the Qinghai-Tibetan Plateau (*Elymus nutans*, *Festuca sinensis*, *Poa pratensis*, and *Vicia unijuga*) either in monocultures or in mixtures. Data were analysed at three scales (at the pot scale, at the monoculture, and at the mixture scale). Results showed that with decreasing patch size, (i) at the pot scale, the seed germination and plant height in both monocultures and mixtures decreased, while the plant biomass in mixtures first decreased and then increased, and the plant biomass in monocultures decreased; and (ii) at the monoculture scale and the mixture scale, the plant height of *E. nutans* in the monoculture first decreased and then increased, while the plant height of the other monocultures decreased. Furthermore, the plant biomass of *E. nutans* in the monoculture first decreased and then increased, while the plant biomass of the rest species combination decreased. This study provides insight into the future restoration of degraded grassland in alpine meadows and the healthy management of artificial grasslands.

## Introduction

Soil resources such as nutrients distribute heterogeneously in natural conditions, and soil heterogeneity is ubiquitous in terrestrial ecosystems (Wei and van Kleunen 2024). Soil heterogeneity significantly affects the structure and function of plant communities (Wijesinghe et al. 2005; García‐Palacios et al. 2012). For instance, it significantly affected the seed germination of plant populations, with higher soil heterogeneity inhibiting plant seed germination (Liu and Hou 2021). In addition, soil heterogeneity could crucially affect the morphological properties of plants such as root length and plant height. For example, Hao et al. (2012) found that the placement of resource-rich patches (centre or edge) significantly influenced the extent of the root system of *Sophora alopecuroides*. Furthermore, soil heterogeneity affected plant biomass, where the medium level of soil heterogeneity supported higher plant biomass (Liu et al. 2021a). Relatively higher level of soil heterogeneity with smaller patch size reduced the seed germination of *Elymus nutans* and *Lolium perenne* (Liu and Hou 2021), while it tends to have no effect on the plant biomass when plants growing in monocultures (Liu et al. 2024). However, these studies mainly focus on the plant populations (Birch and Hutchings 1994; Casper and Cahill 1996; Blair 2001), while plant interactions in plant communities might influence the responses of plant species to soil heterogeneity (Bliss et al. 2002; Hutchings et al. 2003). Therefore, the joint effects of plant interaction and soil heterogeneity on seed germination, plant growth and plant biomass merit further research.

Plant interaction matters in modifying the effects of soil heterogeneity on plants (Mommer et al. 2012). Focal plants are affected by neighbouring plants either positively or negatively, e.g. via competition for resources such as space, light, or nutrients (Chen et al. 2024; Ren et al. 2024; Uyehara et al. 2024), or through positive interactions such as hydraulic lift or microbial enhancement (Yu and D’Odorico 2015; Zhou et al. 2023). Such neighbour effects are crucial in shaping the structure and functioning of plant communities. Previous research found that plant neighbours significantly affected the plant biomass and its allocations, and such effects were dependent on plant functional groups (e.g. grasses and forbs, Liu and Hou 2021; Liu et al. 2021a). Moreover, plant interaction can alter the responses of plant root foraging to soil heterogeneity, and such responses depended on the plant neighbour identity and the soil resource distribution (Mommer et al. 2012; Garlick et al. 2021), and the size of plant individuals (e.g. larger or smaller, Nakamura et al. 2008; Chen et al. 2020). However, the joint effects of plant interaction and soil heterogeneity on the seed germination, plant growth, and plant biomass of plant communities are still unclear.

Accordingly, a manipulation experiment was conducted to explore the joint effects of plant interaction and soil heterogeneity on the seed germination, plant growth, and plant biomass, where four forage species (*Elymus nutans*, *Festuca sinensis*, *Poa pratensis*, and *Vicia unijuga*) dominant in the Qinghai-Tibetan Plateau were grown either in monocultures or in mixtures with two of them growing together, and three levels of soil heterogeneity were developed by varying patch sizes (small, medium, or large). Specifically, we expected that: (i) seed germination of plant communities in pots with smaller patch sizes is assumed to be suppressed as plants tend to reduce their germinations to reduce the negative influences of the strong variation of soil resources in such pots (Liu and Hou 2021). (ii) Plant growth and plant biomass in pots with smaller patch sizes are expected to be promoted as plants growing in the resource-poor patches could absorb soil resources easier from the neighbouring resource-rich patches at a short distance as compared with growing in pots with a larger patch size (Liu et al. 2019). (iii) The abovementioned effects of patch size (soil heterogeneity) should be modified by plant interaction as plant interactions could be positive such as facilitation, negative such as competition for sources or even neutral (Miller and Travis 1996; Callaway 2007).

## Materials and methods

### Experimental design

This experiment was conducted from 1 July to 29 August 2023 in a greenhouse at the Linze Grassland Agriculture Station of Lanzhou University (100° 06′ 04″ E, 39° 11′ 07″ N), in Gansu Province, China. This station is located at an altitude of 1390 m, covers an area of 386.67 hm^2,^ and has a continental, temperate, and dry climate. The annual sunshine duration is 3042 h, and the average annual temperature is 7.6°C. The average annual precipitation and annual evaporation are 121.5 mm and 2337.6 mm, respectively (Hou and Shen 1999).

**Figure 1.**
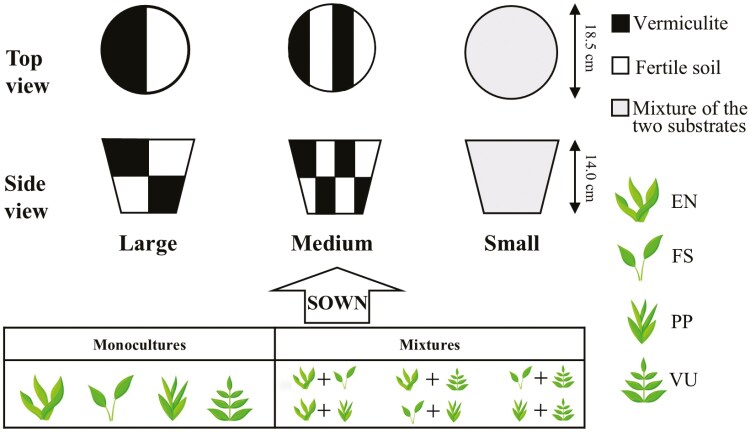
Setup of the experiment, top view and side view of the three levels of soil heterogeneity are presented, with large, medium and small patches arranged from the left to the right. Vermiculite and fertile soil are indicated by black and white colour, while the grey colour represents the mixture of these two substrates. *E*. *nutans*, *F*. *sinensis*, *P*. *pratensis*, and *V*. *unijuga* are labelled as EN, FS, PP, and VU, respectively.

To explore the joint effects of plant interaction and soil heterogeneity on the seed germination, plant growth, and plant biomass, a controlled experiment was done. Accordingly, 3 levels of soil heterogeneity and 10 species combinations were established (Fig. 1), where soil heterogeneity was developed by adopting the idea of Liu et al. (2019). In brief, soil heterogeneity was developed by filling two substrates (vermiculite and fertile soil) strips in an alternate way layer by layer in a pot with a certain patch size. The size of these pots (18.5 cm top diameter, 15 cm bottom diameter, and 14 cm height) was used based on previous research (Li et al. 2021). Note that these two substrates were used to better reflect the different performances of the forage plants growing in each of them. The basic physical and chemical traits of these substrates are listed in Table 1. Ten species combinations were created by growing the four forage plants dominant in the Qinghai-Tibetan Plateau (*E. nutans*, *F. sinensis*, *P. pratensis*, and *V. unijuga*, labelled as EN, FS, PP, and VU, respectively) either in monocultures or in mixtures with two of them growing together. In each pot, 50 seeds of each species in monocultures or 25 seeds of each of the two species in mixtures were uniformly sown. After sowing, the same substrate was added to each patch in all the pots to benefit the seed germination. All seeds in this study were collected from the natural grasslands on the Qinghai-Tibetan Plateau. Thus, there were 30 treatments (3 soil heterogeneity levels × 10 species combinations) in this experiment, and each treatment had five replications, resulting in a total of 150 pots.

**Table 1.** The basic traits of fertile soil and vermiculite applied in this study.

Soil type	Organic matter (g/kg)	N (g/kg)	P (g/kg)	K (g/kg)	pH
Fertile soil	158.70	3.20	0.50	6.06	7.46
Vermiculite	0.00	0.00	0.02	3.71	7.39

All the pots were randomly placed in the greenhouse, which was set at a temperature of 22°C, and no additional lights were added at night. After sowing, water was added at a rate of about 250 ml to each pot every two days. The germination phase of the plants ended when there were no more new seeds germinated in all pots for three consecutive days (Liu et al. 2021b). Plant height in all the pots was measured 3 weeks after the seed germination phase. The plant shoot in each pot was harvested 5 weeks after the seed germination phase by cutting plants in each pot, and roots in each pot were washed out from the soils, and both shoots and roots in each pot were oven dried at 60°C for 48 h and weighed.

Seed germination percentage, plant height, and plant biomass were quantified as follows:

Seed germination percentage = the number of germinated seeds in each pot/the number of added seeds in each pot × 100%.

Plant height was quantified by the average value of plant heights of five plants of each plant species in each pot.

Plant biomass refers to the total biomass including both shoot biomass and root biomass, and it was calculated by dividing the dry biomasses of the total biomass of each species (g) in each pot by the pot area (m^2^).

## Statistical analyses

Analyses were done at three scales, i.e. to test the impacts of patch size, species composition (monoculture and mixture), and their interactions on the plant traits analysis was conducted at the pot scale (including both the monoculture and mixture), to investigate the differences in the responses of plant species to patch size, analysis was done at the monoculture scale (only considering the four plant monocultures), and to explore the effects of patch size that modified by neighbouring identity and plant interaction on the plant traits, analysis was conducted at the mixture scale (only considering the six species mixtures). Specifically, (i) at the pot scale, the general linear model (GLM) was used to explore the effects of patch size, species composition (monoculture and mixture), and their interaction on the seed germination, plant height and plant biomass, where the species combination (four species identities and six species combinations) was treated as a random factor. (ii) At the monoculture scale, GLM was conducted to investigate the effect of patch size, species (*E. nutans*, *F. sinensis*, *P. pratensis*, and *V. unijuga*), and their interaction on the seed germination, plant height, and plant biomass. In order to compare differences in seed germination, plant height, and plant biomass among the three patch sizes, nonparametric tests (Kruskal–Wallis test) were used. (iii) At the mixture scale, GLM was conducted to investigate the effect of patch size, species combination (*E. nutans* + *F. sinensis*, *E. nutans* + *P. pratensis*, *E. nutans* + *V. unijuga*, *F. sinensis *+ *P. pratensis*, *F. sinensis* + *V. unijuga*, and *P. pratensis* + *V. unijuga*), and their interaction on the seed germination, plant height and plant biomass. In order to compare the differences in the seed germination, plant height, and plant biomass among the patch sizes, nonparametric tests (Kruskal–Wallis test) were used. Data were transformed when necessary. All statistics were conducted with SPSS 26.0.

## Results

**Figure 2.**
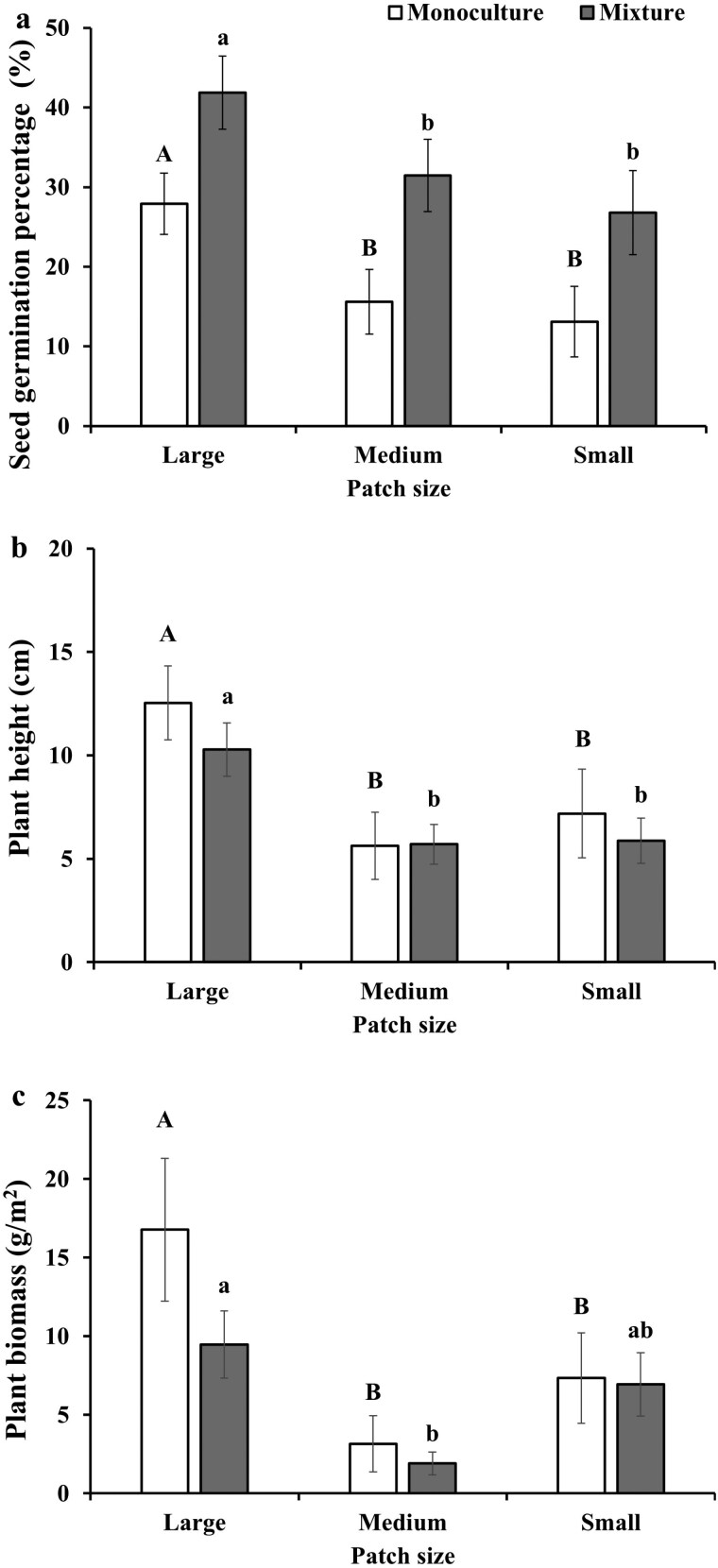
Effects of patch size on the seed germination (a), plant height (b), and plant biomass (c) of monoculture and mixture. Different letters indicate differences of the same species combinations among the patch sizes, and EN, FS, PP, and VU are named as *E. nutans*, *F. sinensis*, *P. pratensis*, and *V. unijuga*, respectively.

At the pot scale, patch size significantly affected the seed germination, plant height, and plant biomass, while species composition (monoculture and mixture) significantly affected the seed germination (Table 2). Specifically, the seed germination and plant height of both monocultures and mixtures decreased along with decreasing patch size (Fig. 2a and b). However, the plant biomass of the monocultures decreased with decreasing patch size, while the plant biomass of the mixtures first decreased and then increased with decreasing patch size (Fig. 2c).

**Table 2.** At the pot scale, effects of patch size, species composition (monoculture and mixture), and their interaction in GLM on the seed germination percentage, plant height, and plant biomass. *F*-values, *P*-values, and degrees of freedom (df) are given, with significant results (*P* < .05) in bold.

Source	df	*F*	*P*
Seed germination percentage			
Patch size	2	5.055	**.008**
Species composition	1	13.134	**<.001**
Patch size × species composition	2	0.029	.971
Plant height			
Patch size	2	8.735	**<.001**
Species composition	1	0.930	.337
Patch size × species composition	2	0.316	.729
Plant biomass			
Patch size	2	9.240	**<.001**
Species combination	1	2.186	.141
Patch size × species composition	2	1.165	.315

**Figure 3.**
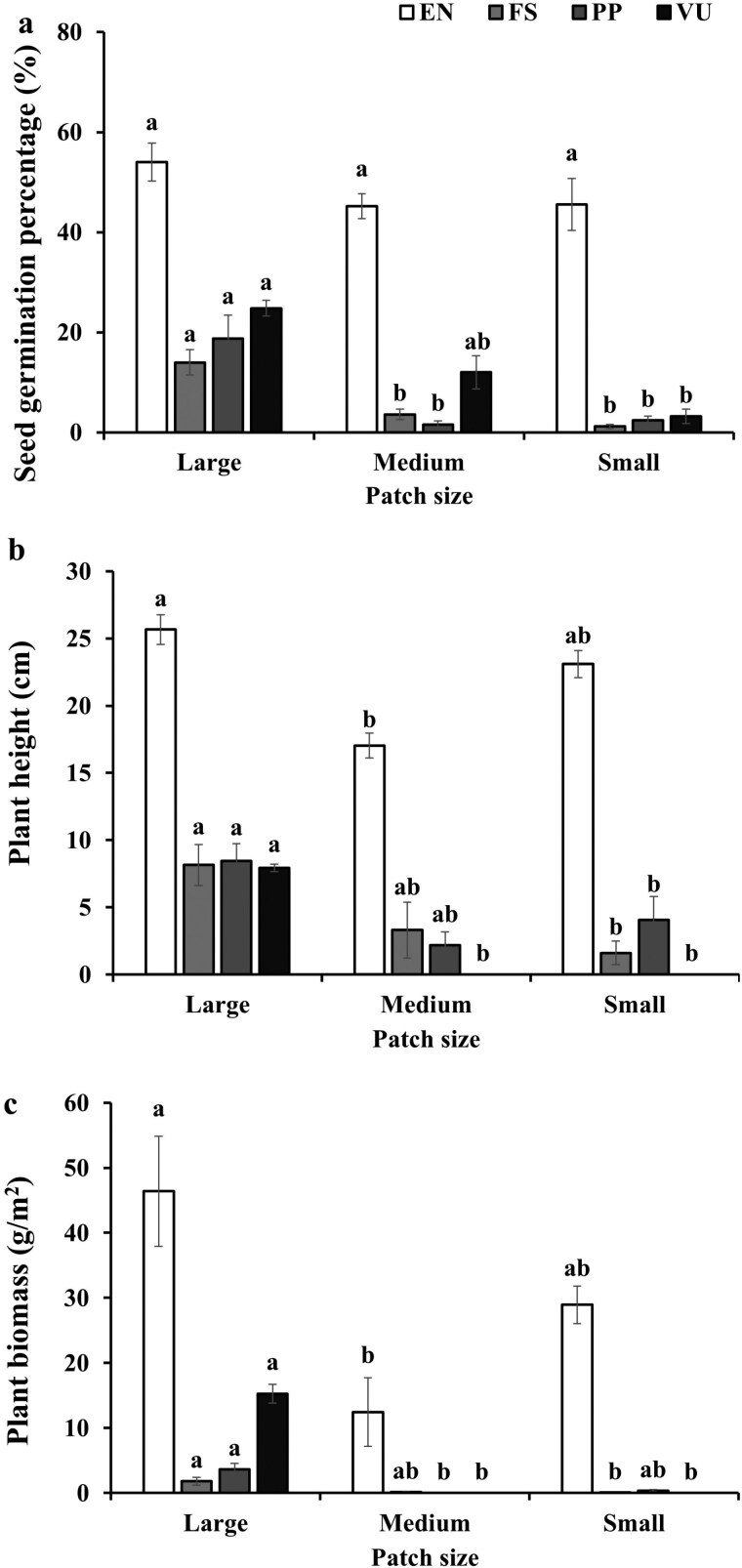
Effects of patch size on the seed germination percentage (a), plant height (b), and plant biomass (c) of monocultures. Different letters indicate differences of the same species among patch sizes, and EN, FS, PP, and VU are denoted as *E. nutans*, *F. sinensis*, *P. pratensis*, and *V. unijuga*, respectively.

**Figure 4.**
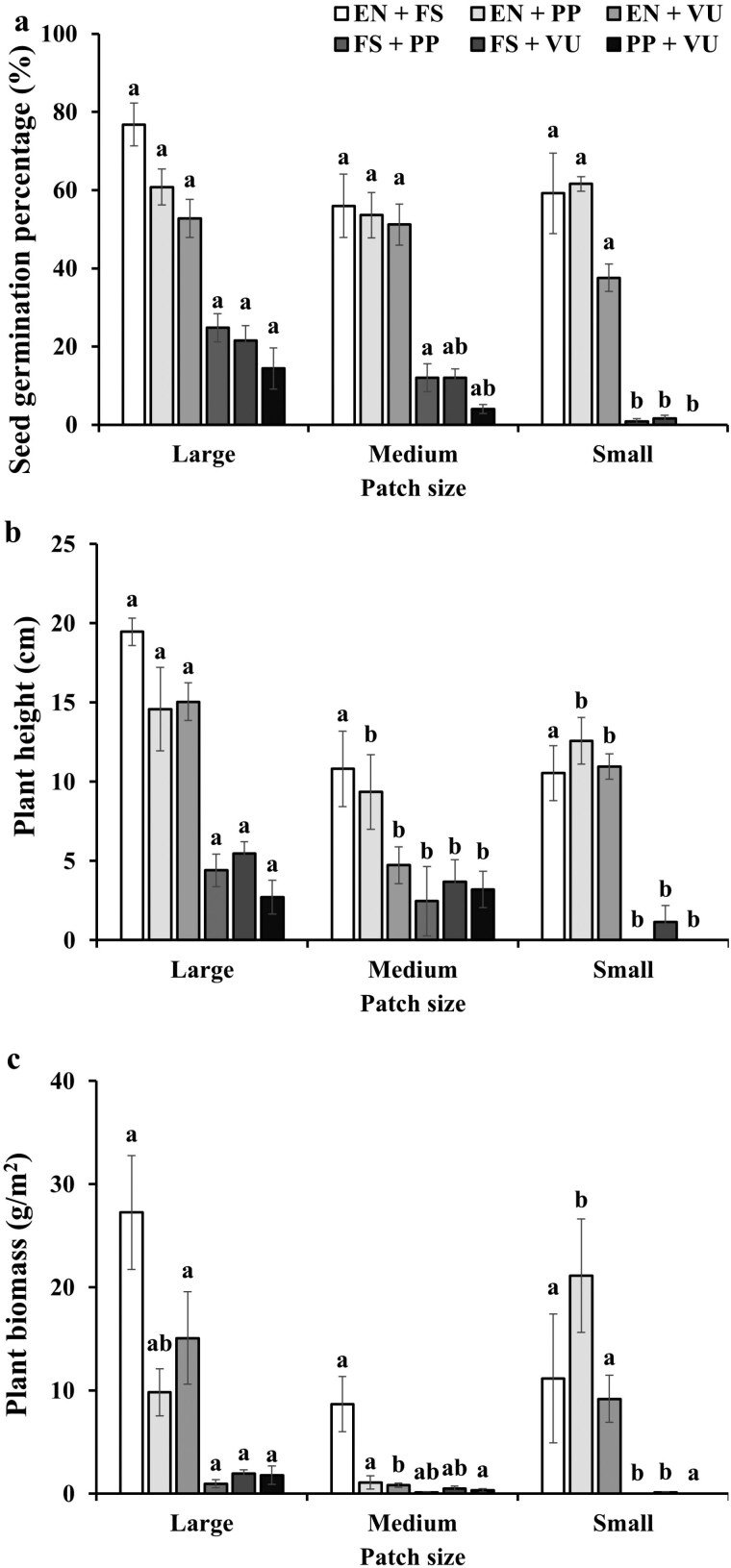
Effects of patch size on the seed germination percentage (a), plant height (b), and plant biomass (c) of mixtures. Different letters indicate differences of the same species combinations among patch sizes, and EN, FS, PP, and VU are labelled as *E. nutans*, *F. sinensis*, *P. pratensis*, and *V. unijuga*, respectively.

At the monoculture scale and the mixture scale, patch size, and species combination significantly affected the seed germination, plant height, and plant biomass, and their interaction significantly affected the plant height and plant biomass (Tables 3 and 4). Specifically, (i) the seed germination of *E. nutans* in the monoculture was not significantly affected by patch size, while the seed germinations of the other monocultures decreased with decreasing patch size (Fig. 3a). Moreover, all the mixtures including *E. nutans* were not significantly affected by patch size, while the seed germinations of the other mixtures decreased with decreasing patch size (Fig. 4a). (ii) Plant height of *E. nutans* in the monoculture first decreased and then increased with decreasing patch size, while the plant height of the other monocultures decreased with decreasing patch size (Fig. 3b). Furthermore, the plant height of the mixture with *E. nutans* and *F. sinensis* was not significantly affected by patch size, while the plant height of the other mixtures decreased with decreasing patch size (Fig. 4b). (iii) Plant biomass of *E. nutans* in the monoculture first decreased and then increased with decreasing patch size, while the rest decreased with decreasing patch size (Fig. 3c). Besides, plant biomasses of the mixtures of *E. nutans* +* P. pratensis* and *E. nutans* +* V. unijuga* first decreased and then increased with decreasing patch size, while the rest decreased with decreasing patch size (Fig. 4c).

**Table 3.** At the monoculture scale, effects of patch size, species (*Elymus nutans*, *Festuca sinensis*, *Poa pratensis*, and *Vicia unijuga*), and their interaction in GLM on the seed germination percentage, plant height, and plant biomass. *F*-values, *P*-values, and degrees of freedom (df) are given, with significant results (*P* < .05) in bold.

Source	df	*F*	*P*
Seed germination percentage			
Patch size	2	25.740	**<.001**
Species	3	121.054	**<.001**
Patch size × Species	6	1.099	.377
Plant height			
Patch size	2	30.962	**<.001**
Species	3	144.018	**<.001**
Patch size × Species	6	1.922	.096
Plant biomass			
Patch size	2	16.858	**<.001**
Species	3	1.922	**<.001**
Patch size × Species	6	5.067	**<.001**

**Table 4.** At the mixture scale, effects of patch size, species combination (*Elymus nutans* + *Festuca sinensis*, *Elymus nutans* *+ Poa pratensis*, *Elymus nutans* *+ Vicia unijuga*, *Festuca sinensis* *+ Poa pratensis*, *Festuca sinensis* *+ Vicia unijuga*, and *Poa pratensis* *+ Vicia unijuga*), and their interaction in GLM on the seed germination percentage, plant height, and plant biomass. *F*-values, *P*-values, and degrees of freedom (df) are given, with significant results (*P* < .05) in bold.

Source	df	*F*	*P*
Seed germination percentage			
Patch size	2	12.895	**<.001**
Species combination	5	73.993	**<.001**
Patch size × Species combination	10	1.136	.348
Plant height			
Patch size	2	14.713	**<.001**
Species combination	5	30.741	**<.001**
Patch size × Species combination	10	2.324	**.020**
Plant biomass			
Patch size	2	9.171	**<.001**
Species combination	5	12.884	**<.001**
Patch size × Species combination	10	3.459	**.001**

## Discussion

Soil heterogeneity significantly affected the seed germination, plant height, and plant biomass (Table 2). Specifically, (i) the seed germination and plant height of both monocultures and mixtures decreased with decreasing patch size (Fig. 2a and b), and such patterns were mainly derived from the monocultures and mixtures without *E. nutans*. (ii) Plant biomass of monocultures decreased with decreasing patch size (Fig. 2c). However, the plant biomass of mixtures first decreased and then increased with decreasing patch size (Fig. 2c), and such pattern mainly derived from the mixtures with *E. nutans*.

According to our first hypothesis, higher seed germination is expected in pots with larger patch sizes. This is confirmed, and the seed germination in both monocultures and mixtures decreased with decreasing patch size (Fig. 2a), i.e. seed germination in both monocultures and mixtures decreased with increasing soil heterogeneity. This is consistent with the finding by Liu and Hou (2021), where the author revealed that seed germination of the plant population in pots with smaller patch size was inhibited. Interestingly, an exception case was found when the analysis was done at the monoculture and the mixture scales, where seed germination of *E. nutans* in both the monoculture and mixtures including *E. nutans* did not vary with patch sizes (Figs. 3a and 4a). However, seed germination of such species in the monoculture was inhibited by patch size in a previous study (Liu and Hou 2021). Such difference might be caused by the different setting of soil heterogeneity in these two studies, where soil patches in Liu and Hou (2021) were filled with fan-shape, while soil patches in this study were filled with strip-shape. Moreover, seed germination percentage in the mixtures was relatively higher than that in the monocultures (Kruskal–Wallis test, *P *= .003). The possible reason might be that plant species in mixtures could germinate at different periods (i.e. early or late) in order to avoid competition, as a result seed germination in the mixtures being higher than that in the monocultures (Gioria et al. 2018).

According to our second hypothesis, plant growth should benefit more when growing in pots with smaller patch sizes. This was not supported as we found that plant height decreased with decreasing patch size (at the pot scale, Fig. 2b) even such pattern was not found in *E. nutans* in the monoculture (Fig. 3b) and in the mixture of *E. nutans* and *F. sinensis* (Fig. 4b). It has been shown that soil heterogeneity influenced the growth rate of plants, consequently affecting the plant height (Hutchings and Wijesinghe 1997). Moreover, plant biomasses of both monocultures and mixtures were affected by patch size (at the pot scale). However, the expected higher plant biomass in pots with smaller patch size was not found both in monocultures and mixtures (Figs. 3c and 4c). Such difference could be caused by the species-specific effects of soil heterogeneity on the morphological traits such as plant height and root length (Stueffer et al. 1996). Moreover, previous research revealed a significant relationship between planting legumes mixed with gramineaes and plant height (Sun et al. 2024). However, the plant height and plant biomass of *E. nutans* mixed planting with *V. unijuga* did not significantly increase in this experiment, which could be related to the allelopathy between *E. nutans* and *V. unijuga* (Chen et al. 2022). Previous research revealed that focal plants could affect its neighbours via allelopathy (Xu et al. 2023; Kong et al. 2024), and the allelopathic effects increased with the distance of the species genotypes (Zhang et al. 2021). Such effects were not measured in this study and merit further research.

According to our third hypothesis, the effects of soil heterogeneity mentioned in the above two hypotheses should be modified by plant interaction. This hypothesis was partly supported as we found that the interaction of patch size × species combination significantly affected plant height and biomass of the mixtures (Table 4). However, such interaction of patch size × species combination was not found in the seed germination (Table 4), even the seed germination of *E. nutans* in the mixture of *E. nutans* and *V. unijuga* was promoted by soil heterogeneity (Supplementary Fig. S1). In other words, seed germination of *E. nutans* in pots with relatively smaller patch size was higher than that in pots with relatively larger patch size. We assume that the substances secreted by *V. unijuga* were affected by patch sizes, as a result influencing the cooccurring species *E. nutans*. Previous research revealed that different plant species growing in heterogeneous soils could interact with each other through varying the speed of movement of plant secretions via a variety of adsorptions and desorptions (Blum 2006).

The results of this work should be interpreted and extrapolated with caution due to the following reasons: (i) Only four forage species were used in this short-term controlled experiment. Further research involving multiple species in a long-term experiment should be considered to better understand the responses of plant communities to soil heterogeneity (Brandt et al. 2013). (ii) The plant-soil feedback-induced soil heterogeneity should be taken into account in the future research as previous studies have indicated that this process is crucial for species selection (Gao et al. 2022), such as the establishment of a ‘fertile island’ by patches of *Achnatherum splendens* (Chen et al. 2005). (iii) Soil heterogeneity affected the retention and transport of allelochemicals and signalling chemicals in soil, which is a determinant for plant–plant interactions (Kong et al. 2024). Thus, to better explore how soil heterogeneity and plant interaction affect the plant community, allelopathic effect should be further considered.

In conclusion, plant interaction and soil heterogeneity significantly affected seed germination, plant height, and plant biomass. Specifically, the seed germination and plant height of both monocultures and mixtures were inhibited by soil heterogeneity, while the plant biomass of mixtures first decreased and then increased, but the plant biomass of monoculture decreased with decreasing patch size. The results of this study provide insight into the future restoration of degraded grassland in alpine meadows and the healthy management of artificial grasslands.

## Supplementary Material

plaf013_suppl_Supplementary_Figure_S1

## Data Availability

The dataset involved in the current study are available with the following link: https://data.4tu.nl/private_datasets/jc_rVOrrO4i2tI4_1D_MS8bv0tewTRYZbM3vbAKFL4Q
