# Supplementary material for: Plant interaction modifies effects of soil heterogeneity on seed germination, plant growth, and biomass of plant communities
Source: AoB Plants. 2025 Mar 8;17(2):plaf013. doi: 10.1093/aobpla/plaf013 (PMC11966607; doi:10.1093/aobpla/plaf013)

Figure S1 Effects of patch size on the seed germination percentage (a), plant height (b), and plant biomass (c) of the monoculture E. nutans, and the effects of patch size and plant interaction on the seed germination (d), plant height (e) and plant biomass (f) of *E. nutans* when growing together with *V. unijuga*. Different letters indicate differences of the same species among patch sizes.

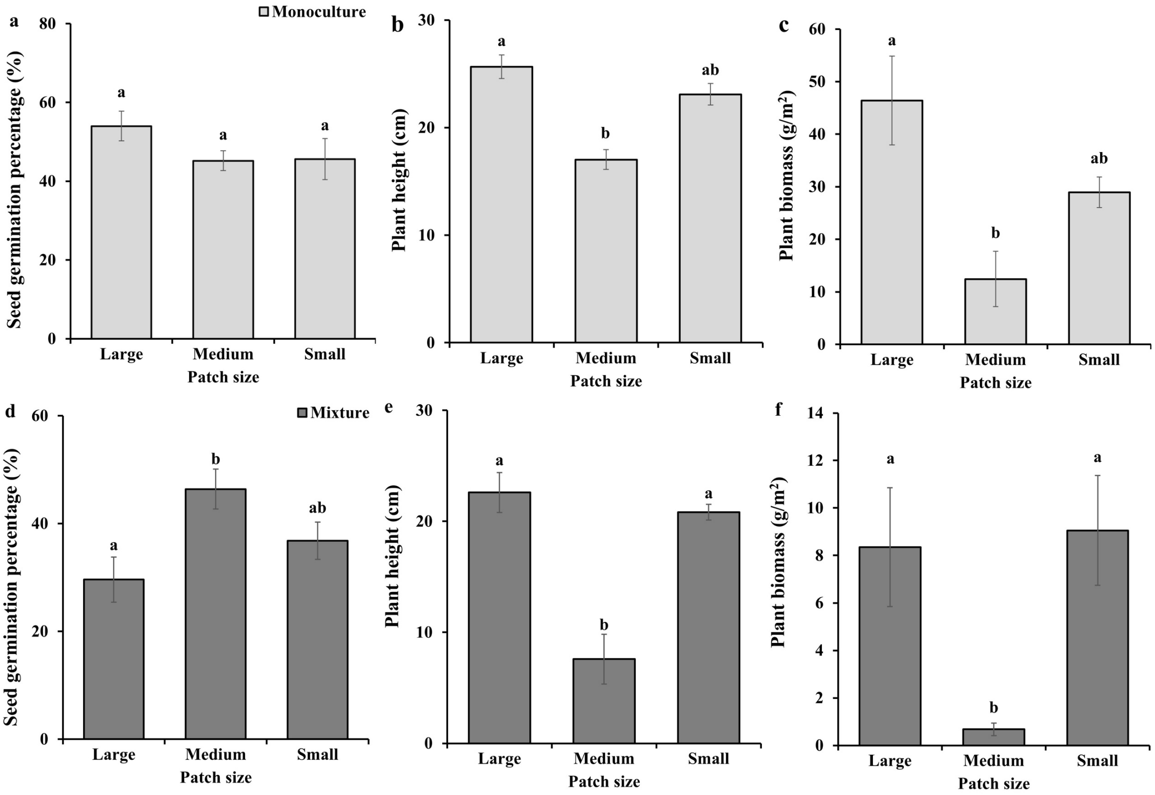

Supplement: plaf013_suppl_Supplementary_Figure_S1 [file plaf013_suppl_supplementary_figure_s1.pdf]
